# Cognitive performance as a behavioral phenotype associated with cocaine self-administration in female and male socially housed monkeys

**DOI:** 10.1038/s41386-024-01882-7

**Published:** 2024-05-17

**Authors:** Mia I. Allen, Marissa B. Costa, Bernard N. Johnson, Robert W. Gould, Michael A. Nader

**Affiliations:** 1 Department of Translational Neuroscience, Wake Forest University School of Medicine, Winston-Salem, NC USA; 2 Center for Addiction Research, Wake Forest University School of Medicine, Winston-Salem, NC USA; 3 Department of Radiology, Wake Forest University School of Medicine, Winston-Salem, NC USA

**Keywords:** Psychology, Neuroscience

## Abstract

Considerable research has suggested that certain cognitive domains may contribute to cocaine misuse. However, there are gaps in the literature regarding whether cognitive performance before drug exposure predicts susceptibility to cocaine self-administration and how cognitive performance relates to future cocaine intake. Thus, the present study aimed to examine cognitive performance, as measured using automated CANTAB cognitive battery, prior to and following acquisition of cocaine self-administration under a concurrent drug vs. food choice procedure in female and male socially housed cynomolgus macaques. The cognitive battery consisted of measures of associative learning (stimulus and compound discrimination tasks), behavioral flexibility (intradimensional and extradimensional tasks), and behavioral inhibition (stimulus discrimination reversal, SDR, and extra-dimensional reversal tasks). After assessing cognitive performance, monkeys were trained to self-administer cocaine (saline, 0.01–0.1 mg/kg/injection) under a concurrent cocaine vs. food schedule of reinforcement. After a history of cocaine self-administration across 3-4 years, the cognitive battery was re-assessed and compared with sensitivity to cocaine reinforcement. Results showed drug-naïve monkeys that were less accurate on the SDR task, measuring behavioral inhibition, were more sensitive to cocaine reinforcement under the concurrent cocaine vs. food choice procedure. Furthermore, following chronic cocaine self-administration, cocaine intake was a negative predictor of accuracy on the SDR behavioral inhibition task. After cocaine maintenance, monkeys with higher cocaine intakes required more trials to complete the SDR behavioral inhibition task and made more incorrect responses during these trials. No sex or social rank differences were noted. Overall, these findings suggest that cognitive performance may influence vulnerability to cocaine misuse. Also, chronic cocaine may decrease levels of behavioral inhibition as measured via the SDR task in both females and males.

## Introduction

Substance use disorders (SUDs) are often characterized by persistent and maladaptive drug use despite negative consequences. In addition to economic and social consequences, drug misuse can also result in deleterious effects to an individual’s physical and mental health [1]. Moreover, overdose deaths have been increasing yearly and cocaine use disorder (CUD) has recently been referred to as the silent epidemic in the United States when compared to the current opioid use crisis [2–4]. Given that there are no FDA-approved treatments for CUD and efficacious behavioral interventions are still limited, there is a vital need for research to examine factors that exacerbate or ameliorate vulnerability to and maintenance of cocaine use. Such research may aid in the development of novel treatments for CUD [5–9].

Considerable research has suggested that cognitive abilities play a crucial role in substance misuse [10–12]. However, it remains unclear if changes in cognitive performance occur due to chronic cocaine exposure or if cognitive differences precede cocaine exposure [13]. For instance, baseline deficits in cognition may increase the probability that an individual first experiments with cocaine and then continues to use cocaine. Bradberry and colleagues conducted longitudinal studies in an effort to begin to address this question. In one study [14], cocaine-naïve monkeys were first trained on two cognitive tasks, a reversal learning task, to measure inhibitory control and a working memory task (delayed match to sample). These tasks were again assessed after 12 months of cocaine self-administration. Cocaine self-administration impaired both cognitive domains; these impairments dissipated after 3 months off from cocaine [15]. Those within-subject studies document cocaine-induced effects on cognition, however, they do not address the issue of predisposition to developing CUD.

Thus, the first goal of the present study was to determine how baseline cognitive abilities, in cocaine-naïve monkeys, would relate to sensitivity to cocaine reinforcement under a concurrent cocaine vs. food choice procedure. These cognitive domains were measured via a battery of assessments including stimulus discrimination and reversal (SD, SDR, respectively), a compound discrimination task (CD), and an attention set-shifting task including an intradimensional shift (ID), an extradimensional shift (ED), and an ED reversal (EDR). Together these six tasks aim to measure associative learning (SD and CD), behavioral inhibition (SDR and EDR), and behavior flexibility (ID and ED).

As mentioned previously, cognitive deficits may also occur due to chronic cocaine use. In fact, research in human subjects has demonstrated that long-term cocaine users show impairments in numerous cognitive domains including attention, memory, inhibitory control, and decision-making when compared to cocaine-naïve individuals [16–18]. Furthermore, cognitive impairments associated with chronic cocaine use have been related to lower rates of treatment initiation and higher treatment attrition rates [19]. Previous research in female cynomolgus monkeys has shown that when compared to drug-naïve animals, monkeys with a cocaine history (mean intake = 753.79 mg/kg) required more trials and made more errors and omissions while learning an associative learning task (SD) and a behavioral inhibition task (SDR) [13]. In another preclinical study, male rhesus monkeys with an extensive cocaine self-administration history (mean intake = 1291.98 mg/kg) required significantly more training trials and made more errors during a reversal learning task and a multi-dimensional discrimination task (ED) when compared to controls [20, 21]. Together, these data suggest that chronic cocaine exposure may impair the ability to learn a novel task requiring associative learning (SD) and behavioral inhibition (SDR) [13, 22]. In addition, chronic cocaine use may impair attentional set-shifting (ED) [20, 22]. A study by Kangas and Bergman, using methamphetamine and squirrel monkeys, also further supported the notion that chronic stimulant exposure may disrupt discrimination learning as measured via an SD task using touch screens [23]. Given these findings, it is possible that chronic cocaine use, by disrupting cognitive abilities, may further perpetuate a cycle of maladaptive drug-use, including the inability to inhibit drug-related behaviors [24]. Thus, the second goal of the present study was to extend earlier research and further characterize how chronic cocaine exposure influences cognitive abilities, specifically associative learning, behavioral flexibility, and behavioral inhibition, using a within-subjects longitudinal design.

In addition to exploring how cognitive abilities predicted vulnerability to cocaine reinforcement and changes following chronic cocaine use, this study also examined whether sex and social rank influenced these outcomes. As it relates to social rank, monkeys living in social groups form hierarchies that are linear and these social hierarchies can be conceptualized as a continuum between chronic social stress and environmental enrichment [25–27]. Additionally, variables related to the organism, such as sex, appear to be crucial factors in influencing drug reinforcement [28]. Despite this, no previous work to our knowledge has examined whether sex and/or social rank influence the relationship between cognitive abilities and cocaine self-administration.

## Methods

### Animals

Twelve cocaine-naïve cynomolgus macaques (6 females and 6 males) served as subjects. All animals were housed in same-sex social groups of 4 and social rank was determined according to the outcomes of agonistic encounters as described previously [29–31]. For the present studies, the #1- and #2-ranked monkeys were considered dominant, and the #3- and #4-ranked monkeys were considered subordinate (Table 1). Not all monkeys from each social group were used in an experiment and the social rank of all twelve animals remained stable throughout the current study. All monkeys had prior behavioral histories involving food-reinforced responding under a concurrent FR schedule of food (1- vs. 3-pellets) reinforcement and delay discounting studies [32]. At the start of this study, all monkeys were naïve to drugs except for infrequent exposure to ketamine (I.M.), used for veterinary or imaging procedures, and 1.5% isoflurane for PET studies [33]. Monkeys in all experiments were housed in a temperature- and humidity-controlled room, maintained on a 14-hour light/10-hour dark cycle (lights on between 6:00 AM and 8:00 PM), in stainless-steel cages. They were fed sufficient standard laboratory chow (Purina LabDiet 5045, St Louis, Missouri, USA) to maintain healthy body weights slightly below free-feeding weights and enriched daily with fresh fruits or vegetables; water was available *ad libitum*, while in the homecage. Environmental enrichment was provided as outlined in the Animal Care and Use Committee of Wake Forest University Non-Human Primate Environmental Enrichment Plan, including chew toys, mirrors, music, and foraging feeders. The menstrual cycle in all females was monitored daily. Animal housing, handling, and experimental protocols were performed per the 2011 National Research Council *Guidelines for the Care and Use of Mammals in Neuroscience and Behavioral Research* and were approved by the Animal Care and Use Committee of Wake Forest University.

**Table 1:** Individual-subject data depicting sex, social rank, cocaine intake at the follow-up CANTAB battery and ED50s on the cocaine-food choice procedure during the initial and secondary determination.

Animal	Sex	Rank	Intake^a^	ED50_1_^a^	ED50_2_^a^
F-8535	Female	Dominant	100.00	0.08	0.003
F-8531	Female	Subordinate	94.10	0.02	0.02
F-8554	Female	Dominant	137.96	0.07	0.02
F-8537	Female	Dominant	95.40	0.01	0.003
F-8557	Female	Subordinate	143.89	0.07	0.02
F-8555	Female	Dominant	37.17	0.004	0.001
M-8562	Male	Subordinate	228.15	0.04	0.02
M-8564	Male	Subordinate	114.99	0.04	0.02
M-8559	Male	Subordinate	139.33	0.07	0.02
M-8558	Male	Subordinate	305.25	0.02	0.02
M-8503	Male	Dominant	324.48	0.006	0.02
M-8502	Male	Dominant	166.90	0.009	0.006

^a^Numbers represent mg/kg.

### General procedures

Each day, monkeys were first studied in cognition assays, followed by food- and/or cocaine-maintained responding in different operant chambers from the cognition studies. Initially, operant responding consisted of only food-maintained responding. After initial cognition assessments, monkeys were permitted to self-administer cocaine (see below) under various schedules of reinforcement [34].

### Catheter implantation

For the self-administration studies, monkeys were surgically implanted with a chronic indwelling intravenous catheter and subcutaneous vascular access port (VAP) under sterile conditions. Details of this surgery can be found in previously published work [35]. All animals were given at least one week to recover from the surgery before experimental sessions began.

### Apparatus

All monkeys, fitted with aluminum collars, were trained to sit in a primate restraint chair (Primate Products, Redwood City, CA). Cognitive testing was conducted using the Cambridge Neuropsychological Test Automated Battery apparatus (CANTAB; Lafayette Instruments, Lafayette, IN) as described previously [13, 22]. Cocaine self-administration experiments were conducted in ventilated, sound-attenuating primate chambers (1.5 × 0.76 × 0.76 m; Med Associates, St. Albans, VT), described previously [35]. During both types of sessions, white noise was played to mask any potentially obstructing sounds from outside the experimental room.

### Cognition training

Drug-naïve monkeys were trained to touch a square that progressively became smaller across trials. Each touch within the square was reinforced with a 190-mg food pellet, followed by a 10-s timeout (TO); touches outside the square resulted in a 10-s TO. Animals were considered trained when for two consecutive daily sessions the monkeys reached the smallest square.

### CANTAB battery

**Fig. 1:**
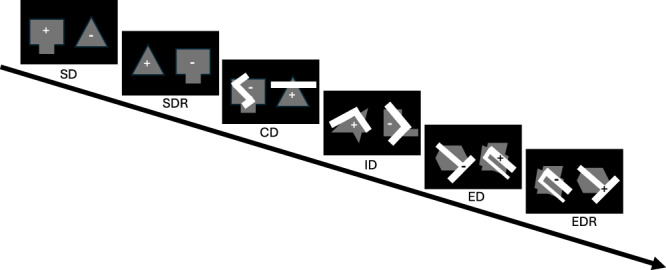
The progression of the CANTAB battery across the six segments (SD, SDR, CD, ID, ED, EDR). Plus signs (+) signify that responding on that shape or line resulted in the dispensing of a food pellet, while minus signs (−) signify that responding did not result in the presentation of a reinforcer and a timeout was implemented.

Once all monkeys were trained to touch the screen, they were trained on six tasks that assessed different cognitive domains (Fig. 1). The first task was a stimulus discrimination (SD) task that assessed associative learning. For this task, two shapes (A, B) appeared in a horizontal row across the center of the screen. A response on one shape (A+) resulted in the dispensing of a 190 mg fruit-flavored food pellet; responses on the other shape (B−) resulted in a 10-s TO. All responses were followed by an additional 7-s inter-trial interval. The shapes were randomly distributed at the two possible locations on the screen and there were 200 maximum trials per session. Successful completion of this segment and all following segments occurred when the animal made 12 correct responses out of the previous 15 completed trials. Upon completion of each segment, the monkeys progressed immediately to the next stage within the same session. If a monkey failed to complete a stage within one session, the next day’s session began at the same stage, with the same stimuli and contingencies from the end of the previous day’s session. Failure to respond within 10 seconds on any trial resulted in termination of the trial and was followed by a 15-sec timeout before the next trial began; these were recorded as omissions. This segment aimed to measure associative learning. Following the SD phase, the reversal learning (SDR) segment began in which responding on the previously correct shape was now incorrect. Responding on the previously incorrect shape (B−) was now counted as a correct response (B+). SDR performance assessed a form of executive function termed behavioral inhibition [21, 22].

After the SDR phase was complete, another form of associative learning was assessed using a compound discrimination (CD) task (see Fig. 1). During this segment, the same two shapes were still present, but lines overlaid the shapes (L1 and L2). These lines were not associated with any reward-related contingencies and instead served as distractors. The same shape that was correct during the SDR phase, remained correct (B+). Although the CD task, like the SD task, measures associative learning, it also involves gating and filtering stimuli given that the animal must now ignore the distracting stimuli. Following successful completion of this segment, two new shapes (C+ and D−) and two new lines (L3 and L4) were introduced but only the shapes continued to be associated with the presentation of the food reinforcer. This stage was called the intra-dimensional (ID) shift stage which aims to measure behavioral flexibility by assessing attentional set-shifting. The next segment in the battery involved an extra-dimensional (ED) shift task which also assesses behavioral flexibility. During the ED task, the previously ignored stimuli (lines) became the stimuli associated with the presentation of the food reinforcer and the stimuli previously associated with the presentation of a food pellet (shapes) become distractors. Again, two new shapes (E, F) were introduced, and two new lines were introduced (L5+, L6−). The final phase was the extra-dimensional reversal (EDR) shift phase where the line previously associated with the reinforcer was now incorrect (L5−) and the previously incorrect line was correct (L6+). Both shapes still served as distractors (E, F). This segment, like the SDR segment, measures behavioral inhibition via reversal learning.

All monkeys completed the battery at least once when they were drug naïve and once after chronic exposure to cocaine. Chronic cocaine exposure was operationally defined as >100 cocaine self-administration sessions. On average these determinations were 3-4 years apart. Total cocaine self-administration sessions at the time of the follow-up cognitive battery were initially assessed but no relationship was found between this variable and any cognitive measures (*data not shown*). Thus, the primary outcome for this study was cocaine intakes (mg/kg). Also, some monkeys had exposure to the battery several times throughout cocaine self-administration and prior to the >100 sessions criterion. The number of times the animals were exposed to the battery ranged from 2 to 5; linear regression analyses found no relationship (*p* > 0.05) between the number of exposures to the cognitive battery and changes in accuracy from the first exposure (*data not shown*). This suggests that any changes in performance from the first determination to the later one were not due to training. For these studies, the first CANTAB battery exposure and the last CANTAB battery determination were used in the analyses.

### Cocaine vs food choice procedure

Following successful completion of the cognitive battery, all monkeys but two (M-8564 and M-8559) were trained to self-administer cocaine under fixed-ratio (FR) 30 and concurrent food vs. drug choice procedures. M-8564 and M-8559 were only trained under a concurrent food vs. drug choice procedure and these data have been published [34]. However, all other animals initially acquired cocaine reinforcement under a simple FR schedule of reinforcement before being transitioned to the choice procedure. There were no relationships between the cognitive variables examined here and cocaine’s potency under the FR schedule of reinforcement (*data not shown*). Furthermore, initial inspection of the data showed that the two males who were not exposed to the FR schedule of reinforcement did not significantly differ from those with experience with both the FR and the concurrent choice procedures in terms of ED50 values (*p* > 0.05) or cocaine intakes (*p* > 0.05). For comparison with cognitive performance, cocaine self-administration (0.003–0.1 mg/kg/injection) under the concurrent schedule was the primary dependent variable (see [35] for a detailed description of the concurrent drug vs. food choice procedure). Briefly, daily sessions, which typically occurred five days per week, ended after 30 choice trials or 60 min had elapsed, whichever occurred first. Most animals completed all available trials each session. Over the course of this study, complete cocaine vs. food choice dose-effect curves were determined at least twice for each monkey.

### Drugs

(-)Cocaine HCl, supplied by the National Institute on Drug Abuse (Bethesda, MD), was dissolved in sterile 0.9% saline. Different doses were studied by changing the drug concentration. All drug doses are expressed as the salt form.

### Data analyses

#### Cocaine self-administration

The cocaine self-administration data from some of these monkeys has recently been published [34]. The primary dependent variable for self-administration presented in this study was total cocaine intake at the time of the cognitive battery and cocaine ED50 values from the cocaine self-administration procedure (Table 1). The cocaine ED50 was calculated from the ascending limb of the concurrent cocaine vs. food dose-response curves and represents the cocaine dose in which choice for both reinforcers was 50%.

#### Cognitive assessments

The primary dependent variables in the statistical analyses for all CANTAB tasks were total trials completed, number of trials correct, total errors, and omissions. Analyses for the entire battery were based on the total number of trials completed across all six tasks, and the average accuracy across all six tasks. Accuracy during the individual tasks of the battery (SD, SDR, CD, ID, ED, EDR) was also assessed. Accuracy was calculated by dividing correct trials by total trials responded and was only calculated when a task was completed. Successful completion of a task occurred when the monkey made 12 correct responses out of the previous 15 completed trials. Mixed-effects ANCOVAs were run to evaluate changes in accuracy on the CANTAB battery across sex and rank. Cocaine intake was used as a covariate in these analyses. Moreover, a mixed-effect ANOVA was run to look at changes in cocaine’s potency during the first and secondary determination with sex and rank as factors. If no significant effects were found with sex and rank included in the model, they were removed, and a paired t-test was run to compare changes in cocaine’s potency.

To determine if baseline cognitive abilities were associated with cocaine potency on a drug vs. food choice procedure (i.e., the ED50 value for cocaine), linear regression analyses were conducted, with sex and social rank as factors. The reference groups (coded as zero in the regressions) were male and dominant monkeys. Cocaine dose-effect curves were determined at least twice in all animals and thus, separate regressions were conducted with ED50 values at the first determination and ED50 values at the second determination. If baseline accuracy on any of the individual components predicted ED50 values, further linear regressions were conducted to explore the number of total trials, omitted trials, correct trials, and incorrect trials in this segment. Given that previous work suggested that chronic cocaine exposure may specifically influence reversal learning, further analyses were run to examine the SDR and EDR segments. To do this, the data were separated into 3 trial bins and the number of bins taken to consistently (at least 3 bins in a row) reach above 50% accuracy on the tasks was analyzed. Several linear regressions were run to determine whether the number of bins required to reach the >50% accuracy criterion at baseline on the SDR and EDR task predicted the potency of cocaine in the self-administration studies.

For the second analyses, linear regressions were conducted to determine if cocaine intake, performance on the first cognitive battery, sex, and social rank predicted performance on the redetermined cognitive battery. Cognitive performance at the first battery was included as a predictor in the model to control for baseline cognitive differences. As with the previous analyses, the reference groups were male and dominant monkeys. If cocaine intake predicted accuracy on any of the individual components, further linear regressions were conducted to explore the number of trials needed to complete the segment, omitted trials, correct trials, and incorrect trials in this segment. Further analyses were also run to examine whether cocaine intake predicted the number of bins needed to reach >50% accuracy on the SDR and EDR segments.

Moreover, linear regressions were conducted to determine whether there were significant effects of sex, social rank, cocaine intake, and cocaine’s potency on several other variables collected during the SDR and EDR segments of the CANTAB battery. These included average latency to respond during each segment and average latency to collect a pellet after selecting the correct stimulus during each segment. No significant effects were found. Averages across the SDR and EDR segments can be found in Supplementary Tables S5 and S6 as a function of determination time, sex, and social rank. For all linear regressions, significance was set at an alpha of 0.05 and all statistical tests were analyzed with SPSS. Furthermore, Bonferroni’s correction was used.

## Results

### Baseline cognitive performance as a predictor for cocaine’s reinforcing potency

**Fig. 2:**
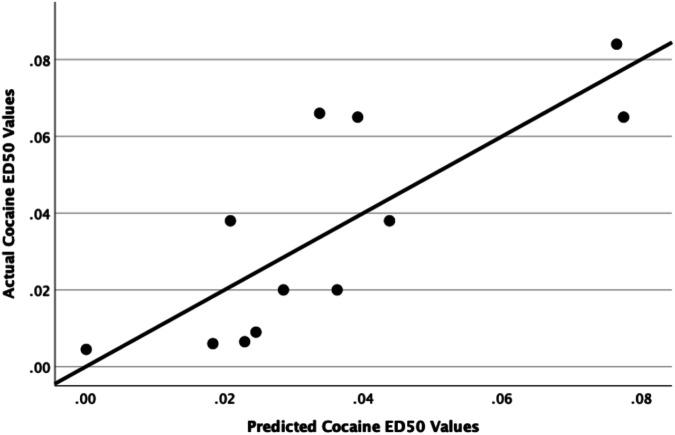
Scatterplot depicting a regression line between actual cocaine ED50 values and predicted cocaine ED50 values for the initial cocaine dose-effect curve determinations based on a model which included SDR accuracy at baseline as well as sex and social rank as predictors.

Percent accuracy on all cognitive tasks ranged from 60 to 80% correct trials, with the lowest performance occurring on the ED and EDR (Supplementary  Fig. S1). Bivariate correlations examining baseline accuracy on each CANTAB segment showed that accuracy only on the SDR and ID tasks were significantly positively correlated (r(10) = 0.693, *p* = 0.018). During the second CANTAB battery determination, there were no significant relationships between accuracy on the segments (*p* > 0.05). Mixed-effects ANCOVAs demonstrated no significant effect of determination time on accuracy on any of the tasks when holding cocaine intake constant (Supplementary Fig. S1). When cocaine self-administration was studied under a concurrent schedule with food pellets as the alternative, the ED50 values for cocaine ranged from 0.005 to 0.08 mg/kg (Table 1 and Supplementary Fig. S2). Linear regressions examining whether baseline cognitive performance predicted cocaine’s potency at the initial determination when cocaine was self-administered under a concurrent schedule, with sex and rank included in the analyses, determined that accuracy on the SDR segment positively predicted ED50 values (t(11) = 3.24, *p* = 0.01) (Table 2 and Fig. 2).

**Table 2:** Linear regressions and significance levels between initial SDR performance and cocaine’s potency during an initial dose-effect curve determination.

Baseline cognitive domain	Standardized B	*P*
SDR accuracy	0.766	0.01^a^
SDR total trials	−2.49	0.04^a^
SDR omitted trials	−0.365	0.31
SDR correct trials	−0.394	0.25
SDR incorrect trials	−0.519	0.13

^a^Asterisks depict significant *p* values.

Further exploration specifically examining the SDR segment determined that accuracy on the SDR segment was correlated negatively to the total trials taken to reach criterion (r(10) = −0.687, *p* = 0.014). Thus, total trials to reach successful completion of the segment was also a significant negative predictor of ED50 values during the initial determination (t11) = −2.49, *p* = 0.037) (Table 2). The number of omitted trials, correct trials, and incorrect trials were not correlated with overall SDR accuracy (*p* > 0.05) and were also not significant predictors of cocaine’s potency during the initial determination when sex and social rank were included in the model (*p* > 0.05). Baseline accuracy on all other segments (SD, CD, ID, ED, EDR) did not predicted initial ED50 values (*p* > 0.05) (Supplementary Table S1). However, the number of bins required to reach >50% accuracy on the SDR and EDR segment at baseline were both significant negative predictors of cocaine ED50 values during the initial determination (t(11) = −0.866, *p* = 0.033 and t(11) = −1.131, *p* = 0.012 respectively) (Table 3).

**Table 3:** Linear regressions and significance levels between the number of 3 trial bins needed to reach >50% accuracy on the SDR and EDR segment and cocaine’s potency during an initial dose-effect curve determination.

Baseline cognitive domain	Standardized B	*P*
SDR bins	−0.87	0.03^a^
EDR bins	−1.13	0.01^a^

Sex and social rank were included in the model.

^a^Asterisks values depict significant *p* values.

A mixed-effects ANOVA evaluating the effect of sex and rank on cocaine’s potency during an initial dose-effect curve determination and a secondary dose-effect curve determination under a concurrent food vs. cocaine choice procedure found a marginally significant effect of determination time on cocaine’s potency (F(1,8) = 4.39, *p* = 0.069) (Supplementary Table S4). However, when sex and rank were not included in the analysis, a paired t-test demonstrated that cocaine’s potency significantly increased at the second determination (t(11) = 2.65, *p* = 0.023) (Table 1 and Supplementary Fig S2). Furthermore, after the second determination, most animals’ cocaine choice dose-effect curve remained stable for the remainder of the study. For the second cocaine potency determination, baseline cognitive performance on all the segments did not significantly predict ED50 values with sex and social rank included (*p* > 0.05) (Supplementary Table S2). In addition, the number of bins required to reach >50% accuracy on the SDR and EDR segment at baseline were no longer significant predictors during the second ED50 determination (*p* > 0.05).

### Cognitive performance as a function of cocaine intake

**Fig. 3:**
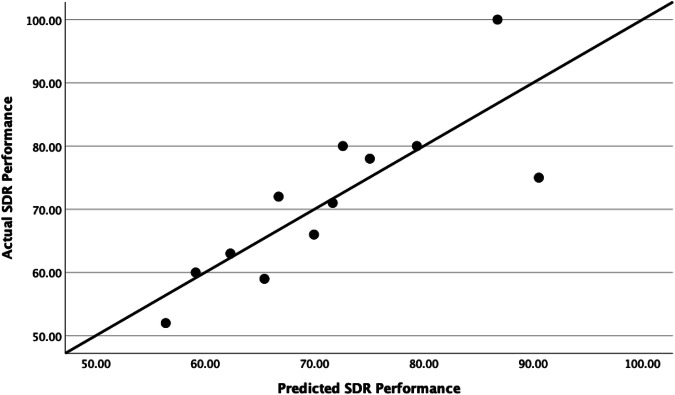
Scatterplot depicting a regression line between actual SDR accuracy and predicted SDR accuracy for the CANTAB determination following chronic cocaine self-administration based on a model which included cocaine intake, SDR accuracy at baseline, sex and social rank as predictors.

Total cocaine intake at the time of the last determination of the cognitive battery varied from 37.17 to 305.25 mg/kg (Table 1). Cocaine intake was significantly negatively related to SDR accuracy during the later determination when sex, social rank, and initial accuracy were included in the model (t(11) = −2.66, *p* = 0.032) (Table 4 and Fig. 3). Further exploration examining the SDR segment determined that overall accuracy was negatively correlated to the total trials to reach criterion (r(10) = −0.685, *p* = 0.014) and the number of incorrect trials (r(10) = −0.810, *p* = 0.001) but not omitted trials or correct trials (*p* > 0.05). Linear regressions demonstrated that cocaine intake significantly positively predicted the total trials needed to successfully complete the segment (t(11) = 3.30, *p* = 0.013). Sex, social rank, and the number of trials needed to complete the segment during the initial determination were also included as predictors in this model. Sex was also a significant positive predictor (t(11) = 2.39, *p* = 0.048) in this model. Although cocaine intake did not significantly predict the number of omitted trials or correct trials during the SDR segment (*p* > 0.05), it did significantly positively predict the number of incorrect trials following chronic cocaine (t(11) = 3.25, *p* = 0.014) (Table 4). Cocaine intake, sex, social rank, and accuracy during the initial assessment did not predict accuracy on the SD, CD, ID, ED, or EDR segments of the battery at the determination following chronic cocaine self-administration (Supplementary Table S3). Sex and social rank were also not significant predictors in any of these models (*p* > 0.05). Additionally, initial performance was not a significant predictor in any of these models suggesting that baseline performance did not influence performance during the follow-up CANTAB battery. In addition, cocaine intake did not predict the number of bins needed to reach >50% accuracy on the SDR and EDR segment (*p* > 0.05).

**Table 4:** Linear regressions and significance levels between cocaine intake and cognitive performance on the SDR task at the re-determination with cognitive performance at baseline, sex and rank included in the model.

Cognitive domain	Standardized B	*P*
SDR accuracy	−0.782	0.03^a^
SDR total trials	1.04	0.01^a^
SDR omitted trials	0.44	0.34
SDR correct trials	0.742	0.07
SDR incorrect trials	0.964	0.01^a^

^a^Asterisks values depict significant *p* values.

## Discussion

Although previous research has shown that social variables [34, 36, 37] and sex [30, 31] influence cocaine self-administration in animals and studies have shown that chronic cocaine impaired cognitive abilities [13–15, 38], no research had directly investigated this relationship in socially housed female and male monkeys. Given this, there is a vital need to further characterize how these variables may influence the relationship between cognitive performance and drug self-administration. Moreover, to our knowledge, no work has elucidated whether cognitive performance prior to any cocaine exposure was associated with the reinforcing potency of cocaine when assessed under a concurrent cocaine vs. food choice schedule of reinforcement. This schedule of reinforcement, commonly used to compare the reinforcing strength of a drug to a non-drug reinforcer, is thought to be highly translational given that in the human population drug use does not occur in a vacuum [39]. Instead, drug use occurs in an environment where an individual must allocate behaviors between drug use and nondrug alternatives.

### SDR task performance and vulnerability to cocaine reinforcement

Using the concurrent cocaine vs. food choice procedure, we demonstrated that baseline cognitive performance on the SDR segment positively predicted cocaine ED50 values during the first dose-effect curve determination. This means that the  monkeys that were more accurate on one of the behavioral inhibition tasks were less sensitive to cocaine reinforcement (i.e., required higher cocaine doses to establish a drug preference) during the initial exposure to the cocaine vs. food choice procedure (see ref. [40]). The significance of this finding suggests a relationship between cognitive performance and protection from cocaine use. Follow-up analyses demonstrated that this effect was primarily driven by the total trials needed to complete the SDR segment; monkeys that required more trials to complete the segment, acquired cocaine reinforcement at lower doses (i.e., cocaine was more potent) compared with monkeys that completed the cognitive task with lower number of trials. In addition, the longer it took a monkey to have an accuracy greater than chance (>50%) on the reversal tasks (SDR, EDR), the more vulnerable they were to cocaine reinforcement.

A major advantage of preclinical research is the ability to study cognitive performance before exposure to cocaine in order to better determine whether a particular cognitive domain is predictive of drug potency and how it changes with long-term cocaine use. In fact, most human-subjects research examining the role of cognition in cocaine misuse has been unable to determine the extent to which cognitive impairments predated or were caused by chronic drug use due to the inherent limitations associated with the study of human subjects [13, 41]. However, one study using the ABCD cohort of human subjects found that lower baseline levels of behavioral inhibition, assessed via self-report on the behavioral inhibition scale (BIS), predicted an increased likelihood of substance use at a 2-year follow-up in a Hispanic population [42]. Similarly to the BIS questionnaire, which primarily measures an individual’s ability to inhibit behavior to avoid punishment, our study supports the notion that deficits in the ability to inhibit behaviors previously associated with a reinforcer and currently associated with a timeout is related to a greater vulnerability to cocaine reinforcement [43]. Given that impaired responding on a reversal task is likely related to deficits in adjusting actions according to changes in environmental contingencies, these impairments may increase susceptibility to maladaptive behaviors [44]. In short, it is possible that low levels of behavioral inhibition, as measured via the SDR task, may increase the probability that a drug reinforcer is chosen even when there is access to an alternative nondrug reinforcer.

The present study, however, did not find a relationship between the reinforcing potency of cocaine under the concurrent schedule of reinforcement and baseline performance on several other cognitive tasks measuring associative learning and behavioral flexibility. Although overall accuracy on the EDR task was not a significant predictor, the monkeys that took more trials to reach an accuracy above chance, tended to have lower ED50 values (i.e., greater reinforcing potency of cocaine). This may suggest that performance specifically on the reversal tasks (i.e., SDR and EDR) may be the most useful behavioral biomarker associated with vulnerability to cocaine reinforcement. However, it is important to highlight the fact that performance on tasks indicated as measuring specific cognitive domains (i.e., SD/CD, SDR/EDR, ID/ED) were not correlated. This suggests that these tasks may be measuring distinctly different components of each cognitive domain. Given this, future work needs to be conducted to explore differences between tasks aimed at measuring the same cognitive domain, as well as how manipulating various variables associated with the CANTAB battery, such as the order of task presentation, influence behavioral outcomes.

Also, the present study only found a relationship between baseline levels of behavioral inhibition as measured via the SDR task and ED50 values during the initial choice dose-effect curve determination. This relationship was no longer detectable following the second dose-effect curve determination which suggests that levels of behavioral inhibition only predict cocaine’s potency during the primary exposure to cocaine under the choice procedure. Thus, it’s possible that behavioral inhibition, as measured via the SDR task, only serves as a predictor for initial vulnerability to cocaine use when there’s an alternative reinforcer available but is not related to the maintenance of cocaine self-administration. Given this, future research should examine whether behavioral or pharmacological interventions aimed to increase behavioral inhibition could decrease vulnerability to cocaine-reinforced behaviors during initial exposure to cocaine when nondrug alternatives are available (see ref. [38]).

### Cocaine intakes and SDR task performance

After long-term cocaine self-administration, there was a direct relationship between cocaine intake and performance on a behavioral inhibition task. That is, greater cocaine intakes were associated with lower levels of accuracy on the SDR task which assessed behavioral inhibition. Further exploration showed that monkeys with higher cocaine intakes tended to take more trials to successfully complete the SDR segment and made more incorrect responses during these trials. Female monkeys also tended to take more trials to complete the SDR segment when compared to males. However, social rank was not a significant variable in any of these analyses. As mentioned previously, past research from our lab has demonstrated that chronic cocaine self-administration disrupted the acquisition of the SD and SDR tasks and resulted in more errors during the ED task [13, 20]. It is important to note that in both of these studies, the monkeys had significantly higher cocaine intakes (mean intakes of 753.79 mg/kg and 1291.98 mg/kg, respectively) when compared to the current study (mean intakes of 157.30 mg/kg, Table 1), suggesting that a broader range of cognitive tasks would be disrupted in the present group of monkeys, as their cocaine intakes increase [11, 17]. In another study, rhesus monkeys exposed to 3.0 mg/kg of cocaine 4 days a week for 9 months showed deficits in their ability to successfully complete a version of the SDR task but not the SD task when compared to control monkeys [45]. While intakes in the above study were most likely higher than in the current study, our findings further support the hypothesis that long-term cocaine use, even at relatively low intakes, disrupts responding on a task that requires an animal to adjust actions according to changes in reward contingencies.

In addition, these data suggest that the SDR task, when compared to the other tasks used in this study, may be the most sensitive to changes following long-term cocaine use since significant effects were only detected in this segment. Although it is unclear why reversal learning was only significantly related to cocaine intake during the SDR task, it’s possible that performance on the EDR task was not significant because the segment was more cognitively challenging and resulted in an overall lower accuracy (Supplementary Fig. S1). However, as mentioned earlier, future work needs to be conducted to elucidate potential differences between the SDR and EDR segments of the CANTAB battery as well as other CANTAB segments that aim to measure the same cognitive domains. Overall, these data support the premise that chronic cocaine self-administration does not disrupt global cognitive functioning but instead selectively influences behavioral inhibition as measured via the SDR task.

The present findings contrast with the results from Kangas et al. [38] in squirrel monkeys. In that study, they found that squirrel monkeys recovered their baseline cognitive performance during chronic cocaine self-administration, while our findings demonstrated that higher cocaine intakes were associated with worse performance on the SDR task. Numerous variables like environmental history, pharmacological history, and experimental design can influence behavioral outcomes. One such difference may have been that the squirrel monkeys were repeatedly exposed (5 times) to the acquisition and discrimination reversal tasks during cocaine self-administration while most of our animals were only exposed to the full CANTAB battery twice (once at baseline and once after chronic cocaine). There are also numerous other important differences such as the animal species used, the housing conditions of the animals, and the cocaine self-administration procedure. In short, an inability to fully replicate the findings in Kangas et al. [38] likely comes from variations in the controlling variables.

### Limitations

There were some limitations to these studies which are important to note. First, this study only examined associative learning, behavioral flexibility, and behavioral inhibition while numerous other cognitive domains also relate to cocaine self-administration [45, 46]. As a result, further research should be conducted to evaluate other subsets of cognition like working memory and attention. Additionally, although this study examined how cognitive abilities relate to cocaine reinforcement under initial exposure to a choice procedure, all animals but two first acquired cocaine self-administration under a simple FR procedure. As mentioned in the Methods, cocaine ED50 values under the FR procedure were not related to any cognitive measures. It is possible though that the relationship between SDR performance and cocaine’s potency under the choice procedure was dependent on the sequence of cocaine self-administration training order in which most animals first acquired cocaine reinforcement when cocaine was the sole reinforcer. Given that in the clinical population cocaine reinforcement is acquired in a context where there are alternative reinforcers, future studies should examine whether the relationships between behavioral inhibition on the SDR task and cocaine’s potency persist when all animals are first exposed to cocaine under a choice procedure instead of an FR procedure. Importantly though, the two animals that acquired cocaine reinforcement only on the choice procedure were not outliers in this study and their data corresponded closely to the other animals (Supplementary Fig. S2). Given this, it’s likely that the effect of training order was minimal on the outcomes noted in this study.

Another limitation is that the reinforcing strength of food was not assessed in these monkeys. It is possible that the animals that had a higher accuracy on the SDR task chose food over cocaine at higher doses because food was more reinforcing in these monkeys. This would suggest that better performance on the CANTAB battery and a lower preference for cocaine on the concurrent drug-food choice paradigm was characteristic of an animal that was more food motivated. This seems unlikely, because one would hypothesize differences across all cognitive domains assessed with food reinforcement. Also, it is possible that chronic cocaine self-administration reduced the reinforcing strength of food which, in turn, resulted in the lower accuracy seen during the SDR segment following chronic cocaine exposure. However, we believe this explanation is unlikely for several reasons. First, we only found significant relationships between CANTAB performance, cocaine ED50 values, and cocaine intake during the SDR segment. If these relationships were due to differences in the reinforcing strength of food, we would expect that all segments of the CANTAB battery would be related to ED50 values and cocaine intakes. Furthermore, other variables such as average latency to respond during each CANTAB segment and average latency to collect a pellet were not significantly related to accuracy on the CANTAB segments, ED50 values or cocaine intakes. This suggests that differences food-maintained responding likely do not account for the relationships seen in this study.

## Conclusions

In conclusion, poorer performance on the SDR task of behavioral inhibition was related to vulnerability to cocaine reinforcement as measured via cocaine’s potency during an initial exposure on the drug vs. food choice procedure. In short, poorer performance on the SDR task was related to cocaine initially functioning as a reinforcer at lower doses which indicated a higher vulnerability to cocaine reinforcement. Furthermore, following long-term cocaine self-administration, lower accuracy on this SDR behavioral inhibition task was associated with higher cocaine intakes. These relationships were independent of social rank and sex, although female monkeys did tend to require more trials to complete the SDR segment after long-term cocaine self-administration. Given these findings, a major hypothesis for future testing is that cognitive performance on the SDR task, measuring behavioral inhibition, represents a behavioral construct that renders individuals more susceptible to initial cocaine use. Additionally, these data provide novel insight into the relationship between cognitive abilities and cocaine self-administration and help inform research examining behavioral and/or pharmacological interventions aimed at reducing cocaine misuse.

## Supplementary information

Supplement

## Data Availability

All data will be made available upon request.
