# Supplementary material for: Cognitive performance as a behavioral phenotype associated with cocaine self-administration in female and male socially housed monkeys
Source: Neuropsychopharmacology. 2024 May 17;49(11):1729–37. doi: 10.1038/s41386-024-01882-7 (PMC11399330; doi:10.1038/s41386-024-01882-7)
Supplement: Supplementary file 1 — Supplement [file 41386_2024_1882_MOESM1_ESM.docx]

**Supplemental tables and figures**

**Table S1**: Linear regressions and significance levels between baseline cognitive performance at the initial determination and cocaine’s potency under the concurrent cocaine-food choice paradigm.

| **Baseline cognitive domain** | **Standardized B** | **p** |
| --- | --- | --- |
| Total trials | -0.297 | *0.41* |
| Average accuracy | 0.372 | *0.29* |
| SD accuracy | 0.123 | *0.75* |
| CD accuracy | 0.503 | *0.13* |
| ID accuracy | 0.755 | *0.12* |
| ED accuracy | -0.023 | *0.95* |
| EDR accuracy | -0.178 | *0.65* |

**Table S2:** Linear regressions and significance levels between baseline cognitive performance at the second determination and cocaine’s potency under the concurrent cocaine-food choice paradigm.

| **Baseline cognitive domain** | **Standardized B** | **p** |
| --- | --- | --- |
| Total trials | 0.040 | *0.89* |
| Average accuracy | -0.074 | *0.80* |
| SD accuracy | -0.020 | *0.95* |
| SDR accuracy | -0.020 | *0.17* |
| CD accuracy | 0.328 | *0.28* |
| ID accuracy | -0.242 | *0.60* |
| ED accuracy | -0.068 | *0.83* |
| EDR accuracy | 0.078 | *0.76* |

**Table S3:** Linear regressions and significance levels between cocaine intake and cognitive performance at the re-determination with cognitive performance at baseline, sex and rank included in the model.

| **Cognitive domain** | **Standardized B** | **p** |
| --- | --- | --- |
| Total trials | -0.084 | *0.85* |
| Average accuracy | -0.131 | *0.75* |
| SD accuracy | 0.180 | *0.62* |
| CD accuracy | 0.454 | *0.09* |
| ID accuracy | -0.265 | *0.88* |
| ED accuracy | -0.225 | *0.68* |
| EDR accuracy | -0.327 | *0.99* |

**Table S4:** Group-subject data depicting the effect of sex and social rank on ED50 initial and secondary determinations. ED50 values depict mean ± SEM.

| **Sex** | **Rank** | **ED50 Determination** | **ED50 Value^¶^** |
| --- | --- | --- | --- |
| Male | Dominant | 1 | 0.006±0.021 |
|  |  | 2 | 0.013±0.005 |
|  | Subordinate | 1 | 0.040±0.015 |
|  |  | 2 | 0.019±0.003 |
| Female | Dominant | 1 | 0.041±0.015 |
|  |  | 2 | 0.007±0.003 |
|  | Subordinate | 1 | 0.042±0.021 |
|  |  | 2 | 0.019±0.005 |

^¶^ numbers represent mg/kg

**Table S5:** Group-subject data depicting the effect of sex and social rank on average latency to respond (ms) and average latency to retrieve a food pellet (ms) during the SDR task. Values depict mean ± SD.

| **Sex** | **Rank** | **SDR Determination** | **Latency to respond** | **Latency to retrieve pellet** |
| --- | --- | --- | --- | --- |
| Male | Dominant | 1 | 1845.6 ± 59.82 | 1578.15 ± 236.81 |
|  |  | 2 | 2871.25 ± 387.57 | 1282.65 ± 132.72 |
|  | Subordinate | 1 | 3071.13 ± 1702.23 | 1358.33 ± 556.72 |
|  |  | 2 | 2711.23 ± 387.57 | 1168.47 ± 731.57 |
| Female | Dominant | 1 | 5351.23 ± 3491.92 | 878.63 ± 401.52 |
|  |  | 2 | 6219.40 ± 4955.21 | 991.20 ± 502.71 |
|  | Subordinate | 1 | 3267.65 ± 2925.37 | 1420.85 ± 45.18 |
|  |  | 2 | 4949.10 ± 1856.16 | 1633.65 ± 376.96 |

**Table S6:** Group-subject data depicting the effect of sex and social rank on average latency to respond (ms) and average latency to retrieve a food pellet (ms) during the EDR task. Values depict mean ± SD.

| **Sex** | **Rank** | **SDR Determination** | **Latency to respond** | **Latency to retrieve pellet** |
| --- | --- | --- | --- | --- |
| Male | Dominant | 1 | 3738.35 ± 472.28 | 1447.20 ± 178.05 |
|  |  | 2 | 3838.05 ± 609.74 | 1254.50 ± 46.10 |
|  | Subordinate | 1 | 4651.30 ± 1009.10 | 1676.40 ± 630.85 |
|  |  | 2 | 5082.30 ± 542.37 | 977.80 ± 442.75 |
| Female | Dominant | 1 | 2424.90 ± 718.02 | 544.70 ± 163.05 |
|  |  | 2 | 2392.10 ± 377.98 | 362.00 ± 110.32 |
|  | Subordinate | 1 | 4756.65 ± 2454.01 | 1593.25 ± 250.95 |
|  |  | 2 | 7318.00 ± 531.32 | 1479.20 ± 466.43 |


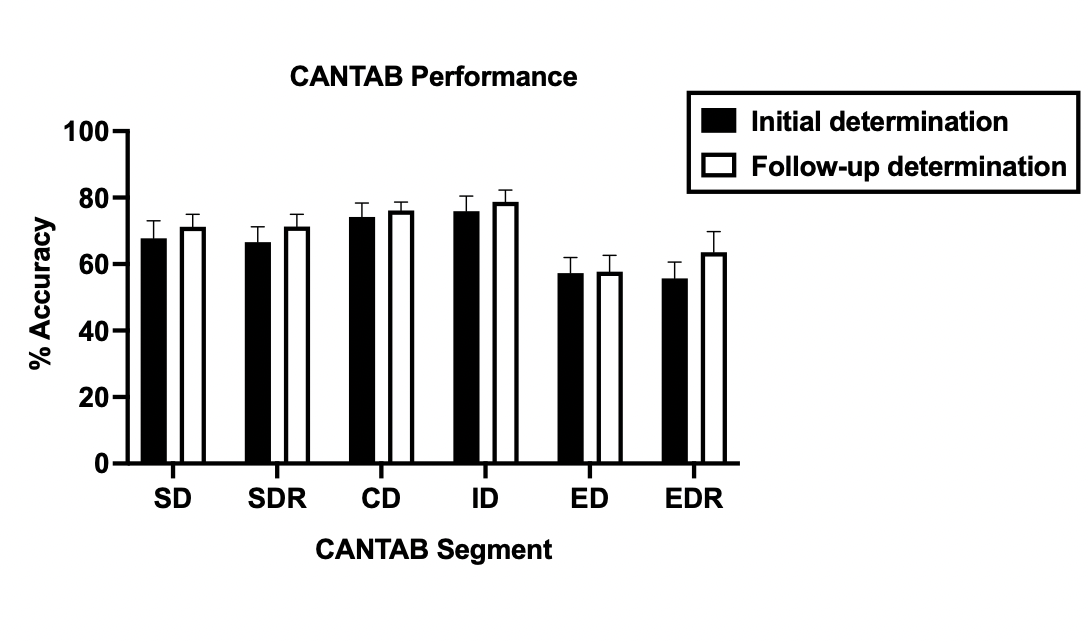


**Figure S1:** Differences in performance accuracy from the initial (black bars) task determination to the follow-up (white bars) determination while holding cocaine intake constant. Each bar represents the mean ± SEM.


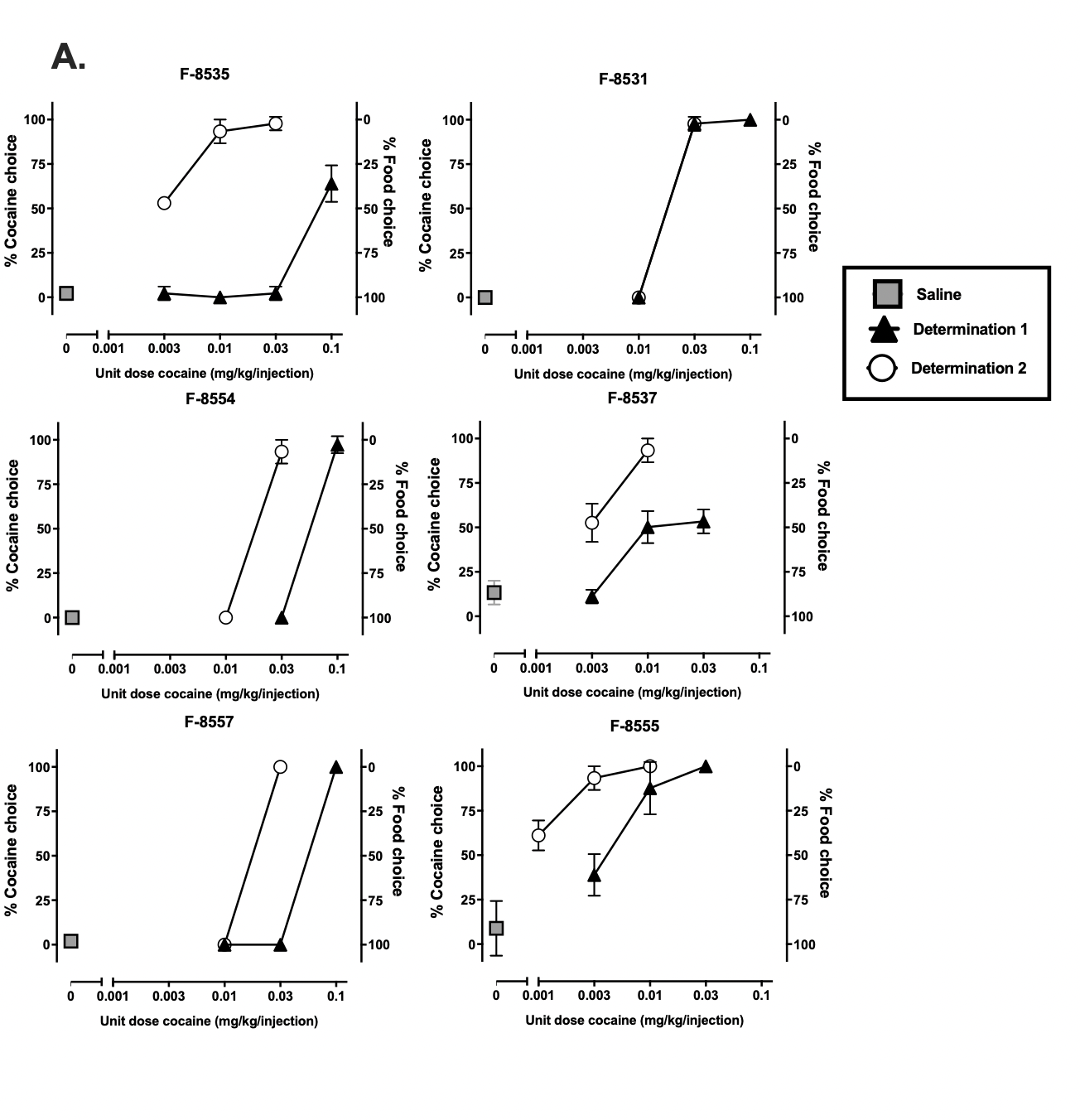


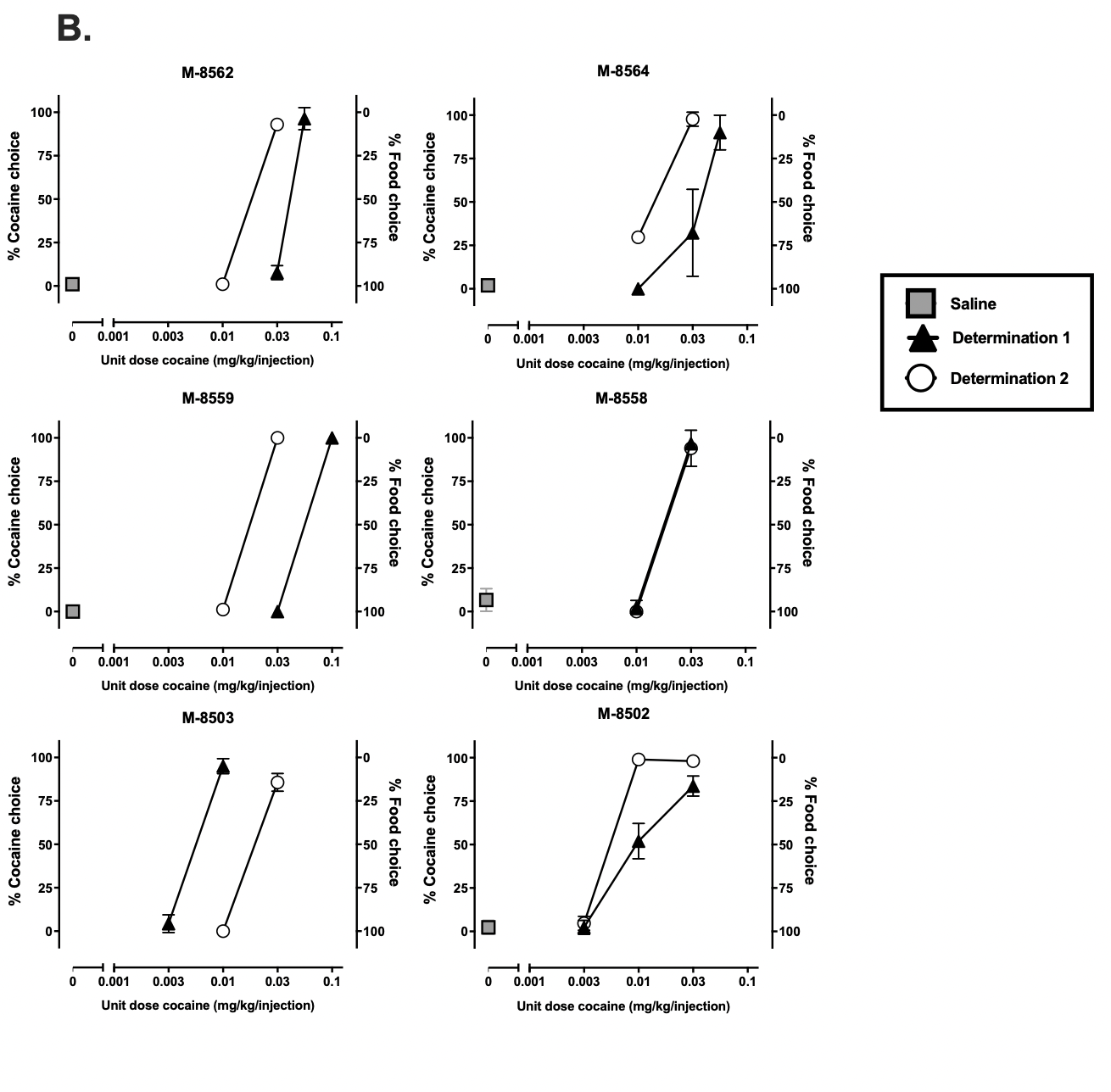


**Figure S2:** Cocaine-food choice dose-effect curves initially (closed triangle) and redetermined (open circle) in individual female (**A**) and male (**B**) monkeys. Each point is the mean ± SD of the last 3 days at each dose. ED50 values (shown in **Table 1**) represents the cocaine dose in which choice for both reinforcers was 50%.
